# Safety and tolerability of single low-dose primaquine in a low-intensity transmission area in South Africa: an open-label, randomized controlled trial

**DOI:** 10.1186/s12936-019-2841-8

**Published:** 2019-06-24

**Authors:** Jaishree Raman, Elizabeth Allen, Lesley Workman, Aaron Mabuza, Hendrik Swanepoel, Gillian Malatje, John Frean, Lubbe Wiesner, Karen I. Barnes

**Affiliations:** 1 Parasitology Reference Laboratory, National Institute for Communicable Diseases, A Division of the National Health Laboratory Services, Johannesburg, South Africa; 2 Wits Research Institute for Malaria, Faculty of Health Sciences, University of Witwatersrand, Johannesburg, South Africa; 3 UCT/MRC Collaborating Centre for Optimising Antimalarial Therapy, University of Cape Town, Cape Town, South Africa; 4 Division of Clinical Pharmacology, Department of Medicine, University of Cape Town, Cape Town, South Africa; 5 Mpumalanga Provincial Malaria Elimination Programme, Mpumalanga, South Africa; 6 UP Institute for Sustainable Malaria Control and MRC Collaborating Centre for Malaria Research, University of Pretoria, Pretoria, South Africa

**Keywords:** Primaquine, Artemether–lumefantrine, Efficacy, Safety, Tolerability, Gametocyte carriage, South Africa, *Plasmodium falciparum*

## Abstract

**Background:**

To reduce onward falciparum malaria transmission, the World Health Organization recommends adding single low-dose (SLD) primaquine to artemisinin-based combination treatment in low transmission areas. However, uptake of this recommendation has been relatively slow given concerns about whether individual risks justify potential community benefit. This study was undertaken to generate comprehensive local data on the risk–benefit profile of SLD primaquine deployment in a pre-elimination area in South Africa.

**Methods:**

This randomized, controlled open-label trial investigated adding a single low primaquine dose on day 3 to standard artemether–lumefantrine treatment for uncomplicated falciparum malaria. Efficacy, safety and tolerability of artemether–lumefantrine and primaquine treatment were assessed on days 3, 7, 14, 28 and 42. Lumefantrine concentrations were assayed from dried blood spot samples collected on day 7.

**Results:**

Of 217 patients screened, 166 were enrolled with 140 randomized on day 3, 70 to each study arm (primaquine and no primaquine). No gametocytes were detected by either microscopy or PCR in any of the follow-up samples collected after randomization on day 3, precluding assessment of primaquine efficacy. Prevalence of the CYP2D6*4, CYP2D6*10 and CYP2D6*17 mutant alleles was low with allelic frequencies of 0.02, 0.11 and 0.16, respectively; none had the CYP2D6*4/*4 variant associated with null activity. Among 172 RDT-positive patients G6PD-genotyped, 24 (14%) carried the *G6PD* deficient (A−) variant. Median haemoglobin concentrations were similar between treatment arms throughout follow-up. A third of participants had a haemoglobin drop > 2 g/dL; this was not associated with primaquine treatment but may be associated with *G6PD* genotype [52.9% (9/17) with A− genotype vs. 31% (36/116) with other genotypes (p = 0.075)]. Day 7 lumefantrine concentrations and the number and nature of adverse events were similar between study arms; only one serious adverse event occurred (renal impairment in the no primaquine arm). The artemether–lumefantrine PCR-corrected adequate clinical and parasitological response rate was 100%, with only one re-infection found among the 128 patients who completed 42-day follow-up.

**Conclusions:**

Safety, tolerability, *CYP2D6* and *G6PD* variant data from this study support the deployment of the WHO-recommended SLD primaquine without *G6PD* testing to advance malaria elimination in South African districts with low-intensity residual transmission.

*Trial registration* Pan African Clinical Trial Registry, PACTR201611001859416. Registered 11 November 2016, https://pactr.samrc.ac.za/TrialDisplay.aspx?TrialID=1859

## Background

It is widely acknowledged that novel tools and strategies are required to eliminate foci of residual *Plasmodium falciparum* malaria transmission [1, 2]. One strategy put forward by the World Health Organization (WHO) is re-purposing primaquine [3–5], the anti-malarial currently recommended for radical cure of relapsing *Plasmodium vivax* and *Plasmodium ovale* malaria [5]. A review of existing data showed single low-dose (SLD) primaquine to be highly effective against mature gametocytes (the natural transmissible parasite stage) [6], thus reducing gametocyte circulation time [7] and malaria transmission to mosquitoes [8]. While artemisinin-based combination therapy (ACT) rapidly clears asexual parasites and early stage gametocytes [9, 10], it is only partially effective against mature gametocytes, allowing gametocytes to persist and remain infectious for up to 14 days after treatment [11], thereby sustaining the transmission cycle [12]. To halt onward *P. falciparum* transmission, the WHO recommended that SLD primaquine be added to the standard ACT treatment in low transmission areas, particularly as a component of elimination or pre-elimination strategies and in areas threatened by resistance of *P. falciparum* to artemisinins [3–5].

Despite the obvious gametocytocidal benefits of primaquine, its uptake has been relatively slow given concerns about whether individual risks justify potential community benefit. Safety concerns relate to haemolytic toxicity seen with the higher primaquine doses required for radical cure of *P. vivax* and *P. ovale* malaria, particularly in glucose-6-phosphate dehydrogenase (*G6PD*)-deficient individuals [13, 14]. This X-linked deficiency increases red blood cell susceptibility to oxidative stress and thus haemolysis, and is prevalent in malaria-endemic tropical and sub-tropical regions as it is protects against severe malaria [15, 16]. Severity of haemolysis depends on the *G6PD* variant present, gender, as well as dose and duration of primaquine exposure [17]. The *G6PD* gene is highly polymorphic, resulting in over 400 *G6PD* variants with enzyme activities ranging from normal to highly deficient [18]. Three variants, B, A+ and A− are most frequently found across sub-Saharan Africa [19]. The wild-type B variant and A+ variant (which carries a single mutation at nucleotide 376), have normal or near-normal enzyme activities. With an additional mutation at nucleotide 202, the A− variant has approximately 12% of the wild type enzyme activity [20] and is generally associated with mild haemolysis [21, 22]. Previous studies have shown the A− variant occurs at frequencies between 2 and 9% in South Africa [23, 24].

The gene coding for the cytochrome P450 2D6 (*CYP2D6*) enzyme, responsible for metabolic activation of primaquine, is highly polymorphic. These polymorphisms are associated with inter-individual variations in the therapeutic efficacy and haemolytic effects of primaquine [25–27] and display marked inter-ethnic frequency differences [28, 29]. The CYP2D6*10 and CYP2D6*17 variants more often detected in Asian and African populations, respectively, are associated with intermediate metabolizer status, while the CYP2D6*4 variant more frequently found in Caucasians is associated with a poor metabolizer status and total loss of *CYP2D6* function in homozygous carriers [29–31]. Previous research has confirmed that the CYP2D6*17 variant is widespread across Africa [32], ranging in frequency from 1 to 33% [33].

With a national malaria incidence of less than one case per 1000 population at risk since the mid-2000s, [34], South Africa officially transitioned to an elimination agenda in 2012 [35]. Although adequate coverage of the existing interventions has largely been achieved [36], residual local transmission persists, impeding the country’s progress towards elimination. The National Malaria Directorate is considering deploying SLD primaquine in these foci of residual transmissions but has expressed concerns over the lack of local data to guide implementation. This study was therefore undertaken to generate comprehensive local data to inform the risk–benefit profile of SLD primaquine deployment in a pre-elimination area within South Africa. Efficacy, safety and tolerability in those randomized to receive primaquine in addition to standard artemether–lumefantrine treatment of uncomplicated falciparum malaria was compared to those randomized to receive standard artemether–lumefantrine treatment alone. Updated information was generated on the prevalence of anaemia and the different *G6PD* and *CYP2D6* variants in the target population. In addition, the impact SLD primaquine had on gametocyte carriage in an area of very low transmission intensity was assessed, as African studies to date have mostly been conducted in areas where more intense malaria transmission facilitates recruitment [37–39]. Lastly, the study also compared lumefantrine concentrations on day 7 between those randomized to receive primaquine and no primaquine, and provided the opportunity to re-assess the therapeutic efficacy of artemether–lumefantrine, which has been first-line treatment in the study area for over a decade.

## Methods

### Study aim, design and setting

This randomized, controlled, allocation-concealed open-label trial investigated the efficacy, safety and tolerability of adding a single low primaquine dose to standard artemether–lumefantrine treatment. The clinical trial was conducted at Komatipoort and Naas primary healthcare facilities that serve a population of approximately 10,000 people in Nkomazi sub-district, Mpumalanga Province, South Africa. During the 2017–2018 financial year the sub-district reported 3438 malaria cases, of which only 626 were classified as locally transmitted. Malaria transmission in the area is unstable and seasonal, occurring predominately during the wet summer months from September to May. Major peaks in transmission are generally observed in January and after Easter, which coincide with increased population movement across the border shared with Mozambique [40]. The predominant malaria parasite is *P. falciparum* with the main vector *Anopheles arabiensis.* In line with national malaria diagnostic and treatment guidelines [41, 42], these nurse-run healthcare facilities offer routine malaria testing using *P. falciparum*-specific malaria HRP2 antigen-based rapid diagnostic test (RDT) kits (First Response^®^, Premier Medical Corporation, India) and treat uncomplicated malaria with artemether–lumefantrine (Coartem^®^, Novartis Pharma, South Africa). Insecticide-based indoor residual spraying is the primary vector control intervention [43].

### Study participants

Individuals aged ≥ 2 years and weighing ≥ 10 kg presenting with a fever (or history of fever in the past 48 h) were screened for eligibility only if they intended to remain in the study area throughout the 6-week follow-up period. When the study health facilities were over-loaded, potential participants were pre-screened for malaria by RDT according to routine practice. Exclusion criteria were relatively strict as primaquine is not currently licensed for use in South Africa, and were: being malaria RDT-negative, evidence of severe illness, and concurrently receiving other drugs that may cause haemolysis, bone marrow suppression or QTc interval prolongation, known allergy to study drugs, any anti-malarial use within the past 4 weeks, blood transfusion within the last 90 days, haemoglobin concentration (Hb) < 7 g/dL, history of haemolysis, rheumatoid arthritis, lupus erythematosus, cardiac disease, and currently menstruating, pregnant or breastfeeding. Prior to being screened for the trial, written informed consent was obtained from all consenting participants aged ≥ 18 years, while consent was provided by parents or guardians of individuals younger than 18 years. Assent was also obtained from children aged ≥ 7 years, with literate witnesses included for patients who could not read.

### Study drugs, randomization and dosing

All enrolled participants were treated with artemether–lumefantrine, administered as the standard 6-dose, weight-based regimen [5, 42]. Participants were given a diary card to record the time of dosing, whether they vomited and what food or drink was taken with each dose. They were encouraged to take all doses with milk or food [44] but were asked to return any doses not taken.

On day 3 participants were randomized in a 1:1 ratio to artemether–lumefantrine alone (standard of care) or artemether–lumefantrine plus primaquine (Primaquine Phosphate Tablets^®^, Sanofi Aventis, South Africa), provided their haemoglobin (Hb) had not decreased by > 2 g/dL from day 0 (unless day 3 Hb ≥ 10 g/dL), and were otherwise eligible to continue. A primaquine target dose of 0.25 mg/kg was administered according to the WHO weight-based dosing recommendations [5]. A randomly-generated sequence of treatment numbers, stratified by clinic, was prepared by an independent statistician using the “rand()” function in Microsoft Excel^®^, who was the only person with access to the randomization schedule and who was not involved in participant assessments. Clinics were instructed to open the next available sequentially-numbered opaque envelope if the patient was eligible for primaquine dosing. Each envelope contained a piece of paper indicating if the participant was randomized to receive primaquine or no primaquine. For children, the dose of primaquine was crushed in water and given in an oral syringe according to the manufacturer-approved extemporaneous preparation procedure. Doses of artemether–lumefantrine and primaquine were repeated if the participant vomited within 30 min, and participants withdrawn if vomiting persisted thereafter.

### Clinical procedures

Participants were asked about previous and current medical conditions during screening, and use of medicines (allopathic, traditional, complementary) throughout the study. Demographic data including age, gender, body weight, occupation, current area of residence, travel history, and country of origin were collected at screening, while vital signs (tympanic temperature, pulse rate, blood pressure and respiratory rate) were recorded at each visit. A physical examination was conducted at baseline as per the standard of care. Thereafter, any physical examinations were symptom-directed. Staff were specifically re-trained in potential signs and symptoms of severe malaria and haemolysis.

As the type of questioning can influence the data collected [45, 46], participants were asked about their health during the study according to a standard study-specific practice, to elicit participant-reported adverse events (AEs). Severity of AEs except fever were classified as: mild (awareness of symptoms that are easily tolerated and do not interfere with usual daily activity); moderate (discomfort that interferes with or limits usual daily activity); or severe (disabling, with subsequent inability to perform usual daily activity, resulting in absence or required bed rest). Fever was categorized as mild (37.5–38.0 °C); moderate (> 38.0 to 39.0 °C); or severe (> 39.0 °C). Seriousness of an AE was categorized as per ICH E2A [47], with a Hb drop of ≥ 40% of baseline Hb and/or requiring a blood transfusion, and/or Hb values of ≤ 5 g/dL included as serious adverse events (SAEs). The relationship of AEs with primaquine was assessed as not related, unlikely, possible, probable and unassessable/unclassifiable. All AEs were then coded using the Medical Dictionary for Regulatory Activities (MedDRA) terminology, version 20 [48].

Haemoglobin level was assessed at each visit by finger-prick blood sample using a HemoCue^®^ photometer (Ängelholm, Sweden) and haemoglobinuria tested for with a urine dipstick. G6PD enzyme activity was assessed for all enrolled participants using the G6PD Biosensor Analyser (Care Start, AccessBio, New Jersey, USA).

On day of enrolment and each subsequent visit (scheduled or unscheduled), duplicate thick and thin blood smears and three dried blood spots (DBS) on Munktell TFN filter-paper cards (Munktell, Germany) were collected. One set of thick and thin smears were sent directly to the Mpumalanga Provincial Malaria Laboratory for staining and reading, to inform eligibility and clinical management. The other set was couriered with the individually packaged DBS to the Parasitology Reference Laboratory at the National Institute for Communicable Disease (NICD) for analysis. On day 7 an additional three 50 µL dried blood spots were collected via lithium heparin microcapillary tubes onto Whatman 31ET CHR filter paper pre-treated with tartaric acid for lumefantrine concentration assays at the University of Cape Town (UCT).

### Microscopy

Malaria microscopy was performed according to national malaria diagnostic guidelines [41]. Asexual parasites were counted against 200 white blood cells (WBC) for high parasitaemias (≥ 100 parasites observed) or 500 WBC for low parasitaemias (< 100 parasites observed). A slide was considered negative if no parasites were observed after 200 fields had been examined. Every slide was read by two independent microscopists. If the parasite densities differed by more than 25%, or if there were discordant results, an additional reading was performed by a third independent microscopist. An average of the two closest readings was taken as the final result.

### Molecular analysis

Parasite RNA was extracted from the filter-paper blood samples using the Qiagen RNeasy mini extraction kit (Qiagen, Germany). Gametocyte carriage was assessed using the reverse transcriptase-polymerase chain reaction (RT-PCR) method to detect pfs25 transcripts with a detection limit of 1–2 gametocytes/µL as described by Mlambo et al. [49].

The Qiagen DNA mini extraction kit (Qiagen, Germany) was used to extract parasite and human DNA from the filter-paper blood samples. Once confirmed as *P. falciparum* by multiplex polymerase chain reaction (PCR) [50], polymorphism analysis of *kelch13*, *crt* and *mdr1* genes was conducted. The propeller domain of the *kelch13* gene was amplified using the protocol of Talundzic et al. [51] and subjected to Sanger sequencing. Sequences obtained were aligned against a reference *P. falciparum kelch13* gene (XM_001350122.1) using a BLAST search and BioEdit Software to identify 25 specific alleles selected according to their association with prolonged parasite clearance half-lives [52]. Primers, PCR conditions and restriction endonucleases used to detect polymorphisms in the *mdr1* (codon 86) and *crt* (codon 76) genes have been described previously [53, 54]. The *mdr1* gene copy number was assessed using a previously described qPCR method [55]. Multiplicity of infection was determined using the protocol of Ranford-Cartwright et al. [56].

*G6PD* variant genotyping to detect the most common African variants, A+, A− and B [19] was performed using a previously published protocol [19]. Genotyping of the CYP2D6*4, 10* and *17 variants was conducting using the protocol of Naveen et al. [29]. A subset of samples was sent for sequencing to validate the PCR–RFLP protocols.

### Lumefantrine concentration analysis

Pharmacokinetic assays were performed using a modification of the method developed by Blessborn et al. [57]. The assay was optimized and validated at the UCT Division of Clinical Pharmacology Analytical Laboratory. The lower limit of quantification was 0.0391 µg/mL.

### Data management and statistical analysis

Assuming a 10% loss to follow-up, a sample size of 70 participants per arm will provide over 80% power at the 0.05 significance level to detect at least a 50% reduction in RT-PCR gametocyte prevalence between study arms (e.g. 40% vs. 20% on day 7). This sample size would also be sufficient to detect a clinically significant (2 g/dL) decrease in mean Hb between study arms (e.g. 11 g/dL vs. 9 g/dL). Although this sample size calculation assumed a slightly lower RT-PCR gametocyte baseline prevalence and smaller primaquine effect than Gerardin et al. [10], the low prevalence of gametocytes from day 3 onwards precluded the assessment of efficacy in this study.

Clinical study data were collected using paper-based source documents, 100% of which were monitored for missing, unexpected and out-of-range dates and values. These data were entered into a REDCap electronic data management system hosted at UCT [58]. Source data verification was performed on 100% of the electronic data by the study team and a random sample of 10% by the trial sponsor; the UCT Clinical Research Centre sponsored this investigator-initiated study. The database was locked, and de-identified electronic datasets were exported to STATA version 15.1 (StataCorp, College Station, Texas) for analysis once all outstanding data queries had been resolved. Molecular drug resistance marker, parasite, gametocyte, *G6PD* and *CYP2D6* data from the NICD were transferred to UCT as MSExcel worksheets for merging with the clinical STATA file. Although the translation of genotype information into metabolizer phenotype is challenging given the range of activity possible for each *CYP2D6* allele, an ‘activity score’ (AS) was inferred from each *CYP2D6* variant as described by Gaedigk et al. [59]. Briefly, the AS represents the sum of values assigned to each individual allele according to their perceived function: non-functional alleles (CYP2D6*4) were given an AS value of 0, reduced-function alleles (CYP2D6*10 and CYP2D6*17) an AS value of 0.5 and functional alleles (CYP2D6*1 or wild-type) an AS value of 1. Those with an activity score or 2.0 or 1.5 could be considered extensive metabolizers (EM), an activity score or 1.0 or 0.5 intermediate metabolizers (IM), and an activity score of 0 poor metabolizers (PM) [30].

## Results

### Baseline characteristics

**Fig. 1:**
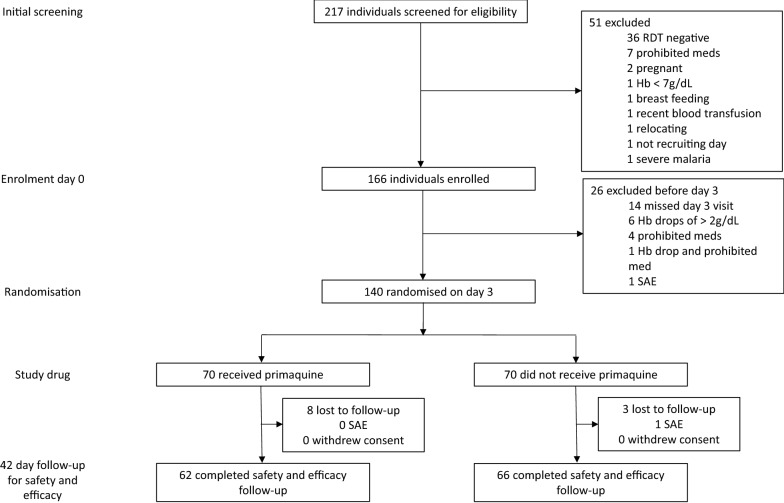
Trial profile and patient disposition. *SAE* serious adverse event, *Hb* haemoglobin

Between 14 December 2016 and 7 June 2018, 217 patients with suspected malaria were screened. Of the 181 malaria RDT-positive patients, 166 met the study criteria and were enrolled. Of these, 140 were randomized on day 3, 70 to each study arm. A total of 128 (91%) participants, 62 (89%) in the primaquine arm and 66 (94%) in the no primaquine arm, completed follow-up until day 42 (Fig. 1).

Table 1 summarizes baseline characteristics for all screened who were malaria RDT-positive (considered representative of the target population) and for those randomized to primaquine or no primaquine. Baseline characteristics of the participants in both treatment groups were similar for age, gender, bodyweight, haemoglobin, and asexual and gametocyte parasite density by microscopy. However, there was a higher prevalence of individuals carrying multiple *P. falciparum* clones (p = 0.042) and gametocytes by PCR (p = 0.03) in the primaquine arm compared to the no primaquine arm. The median (IQR) primaquine dose administered in those randomized to primaquine was 0.25 (0.24–0.27) mg/kg.

**Table 1:** Patient characteristics at baseline and randomization

	Total RDT positives screened [n = 181]	Randomized to primaquine [n = 70]	Not randomized to primaquine [n = 70]
Age (years)	32.1 (24.8–38.7)	32.2 (24.8–39.6)	32.4 (24.3–38.8)
Gender, female n (%)	55 (30.4)	22 (31.4)	16 (22.9)
Bodyweight (kg)	60 (55–64)	60 (53–63)	60 (55–64)
Day 0 haemoglobin (g/dL)	12.9 (11.3–14.2)	13.0 (11.6–14.5)	13.3 (11.6–14.5)
Day 0 anaemia (< 10 g/dL)	14/177 (7.9)	4/70 (5.7)	3/69 (4.4)
Day 3 haemoglobin (g/dL)	12.5 (11.2–13.7)	12.4 (11.3–13.9)	13.0 (11.7–13.6)
Day 3 anaemia (< 10 g/dL)	18/150 (12.0)	5/70 (7.1)	7/70 (10)
Day 0 asexual parasite density/per µL, geometric mean (95% CI)	5554 (3971–7768)	5048 (2933–8688)	4769 (2782–8175)
Day 0 multiplicity of infection (2 or more clones detected)	121/165 (73.3)	41/65 (63.1)	50/63 (79.4)
Day 0 gametocyte prevalence by microscopy	5 (2.3)	3 (4.3)	2 (2.9)
Day 0 gametocyte prevalence by PCR	109/176 (61.9)	48/69 (57.8)	35/68 (42.2)

Categorical variables summarized as number (%); continuous variables summarized as median (IQR), unless otherwise stated

### *G6PD* variant prevalence

Among the 172 RDT-positive patients *G6PD*-genotyped, 24 (14%) carried the *G6PD* deficient (A−) variant, with the remainder of the participants carrying the *G6PD* variants associated with normal enzyme activity [B variant, 110/172 (64%)] or mild deficiencies [A+ variant, 38/172 (22%)], as shown in Table 2. Of the 24 patients with A− genotype, 17 were hemizygous males and 7 were heterozygous females. There was good correlation between the *G6PD* phenotypic screening data generated by the CareStart^®^ G6PD Biosensor and the genotypic data (Kruskall–Wallis p = 0.018). Biosensor readings of below 30 were observed more frequently in individuals carrying the A− variants than other genotypes [7/22, (31.8%) vs 16/138 (12.2%); p = 0.012]. The higher prevalence of the A− variant among those randomized to primaquine (11/66, 18%) than those not randomized to primaquine (6/67, 10%) had no statistical significance (p = 0.183).

**Table 2:** Glucose-6-phosphate dehydrogenase (G6PD) genotype and phenotype and cytochrome P450 2D6 (CYP2D6) genotype and activity scores among RDT-positive participants screened and randomized; n (%)

	Total RDT positives screened [n = 181]	Randomized to primaquine [n = 70]	Not randomized to primaquine [n = 70]
*G6PD* variant genotype			
A−	24 (14.0)	11 (16.7)	6 (9.0)
A+	38 (22.1)	9 (13.6)	17 (25.4)
B	110 (64.0)	46 (69.7)	44 (65.7)
G6PD phenotype (U/dL)	73 (51–91)	68 (51–89)	74 (40–90)
*CYP2D6* variant genotype (activity score, AS^a^)			
*1/*1 (AS 2.0; EM)	129 (74.4)	48 (71.6)	49 (74.2)
*1/*10 (AS 1.5; EM)	13 (7.6)	5 (7.5)	5 (7.6)
*1/*17 (AS 1.5; EM)	18 (10.5)	8 (11.9)	8 (12.1)
*1/*4 (AS 1.0; IM)	1 (0.6)	1 (1.5)	0 (0)
*10/*17 (AS 1.0; IM)	3 (1.8)	2 (3.0)	0 (0)
*17/*17 (AS 1.0; IM)	4 (2.3)	2 (3.0)	2 (3.0)
*4/*10 (AS 0.5; IM)	1 (0.6)	0 (0)	1 (1.5)
*4/*10/*17 (AS 1.0; IM)	2 (1.2)	1 (1.5)	1 (1.5)

^a^CYP2D6 activity score is the sum of the per-allele scores; a null allele having a score of 0, a deficient allele a score of 0.5 and a normal allele a score of 1. CYP2D6 poor metabolizers (PM AS = 0), intermediate metabolizers (IM AS 0.5–1.0), extensive metabolizers (EM AS 1.5–2.0) [30, 31]

### CYP2D6*4, CYP2D6*10 and CYP2D6*17 variant prevalence and phenotype inference

Overall, among the 171 malaria RDT-positive patients genotyped, prevalence of the CYP2D6*4, CYP2D6*10 and CYP2D6*17 mutant alleles was low with allelic frequencies of 0.02, 0.11 and 0.16, respectively (Table 2). The majority [129/171 (75.4%)] had an activity score of 2, with an activity score of 1.5 in 31 (18.1%) participants, an activity score of 1 in 10 (5.9%) participants, and an activity score of 0.5 in 1 participant (0.6%). None had the CYP2D6*4/*4 variant associated with null activity. Two subjects had heterozygous genotypes for all 3 variants tested (*4/*10/*17), similar to *CYP2D6* genotypes previously described by Montané Jaime et al. [60].

### Gametocyte carriage

Gametocyte carriage detected by microscopy and PCR on day 0 differed markedly, as expected, with PCR detecting over 15-fold more gametocytes than microscopy (Table 1). By day 3, prior to randomization and primaquine administration, gametocyte carriage had decreased substantially with only two individuals, both in the primaquine arm, with gametocytes detected by PCR. No gametocytes were detected by either microscopy or PCR in any of the follow-up samples collected after randomization (from day 7 until day 42), precluding an assessment of the effect of primaquine on gametocyte carriage.

### Haematological response

**Fig. 2:**
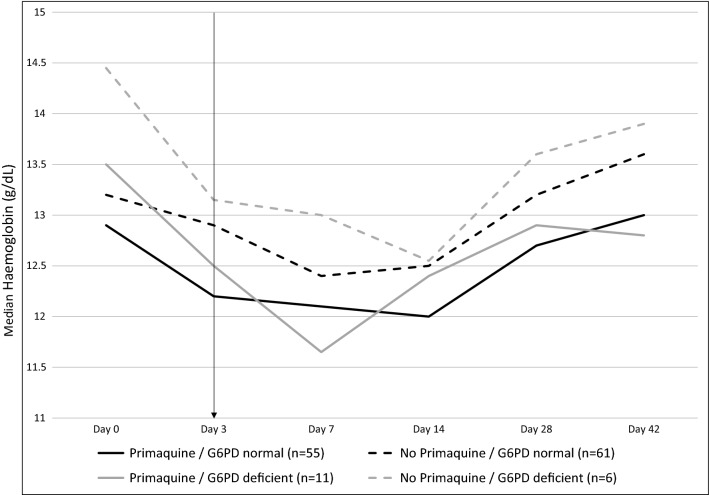
Median haemoglobin (g/dL) concentrations over time, by treatment arm (artemether–lumefantrine plus primaquine (solid lines) and artemether–lumefantrine alone (No Primaquine—dashed lines)) and G6PD status (G6PD normal (B or A+ variant—black lines) and G6PD deficient (A− variant—grey lines) with the arrow indicating day 3 when randomization and primaquine treatment occurred. G6PD: glucose-6-phosphate dehydrogenase

On day 3, haemoglobin concentrations among those enrolled but not randomized were significantly lower (Kruskall–Wallis p < 0.0001), and the risk of anaemia significantly higher (6/10 vs 12/140; p < 0.001), compared to the 140 participants randomized, reflecting the haemoglobin-based randomization criteria. Among those randomized, median Hb was slightly lower in the primaquine arm compared to the no primaquine arm, both before (day 0 and 3) and after (days 7–42) randomization and primaquine dosing (Fig. 2). These differences were however not statistically significant. The median Hb nadir occurred in both arms on day 7 (Fig. 2). One third of participants had a drop in Hb from baseline of > 2 g/dL, but this was not associated with primaquine treatment [24/70 (34%) in the primaquine arm and 23/69 (33%) in the no primaquine arm]. However, this drop in Hb may be associated with *G6PD* genotype (Fig. 2), as 52.9% (9/17) of participants with the A− genotype experienced such a drop in Hb compared to 31% (36/116) of participants with other genotypes (p = 0.075). Anaemia (defined as Hb < 10 g/dL) was present at baseline in 4 individuals in the primaquine arm and 3 participants in the no primaquine arm, and emerged during follow-up in 6/66 (9.1%) in the primaquine arm and 11/67 (16.4%) in the no primaquine arm (p = 0.21). If the WHO standard (< 10.9 g/dL for non-pregnant patients aged > 5 years [61]) rather than local current standard of care (< 10 g/dL) was used for the definition of moderate/severe anaemia, anaemia was more prevalent at each follow-up visit but remained similar between treatment arms.

### Adverse events

A total of 74 AEs, other than the anaemia described above, occurred in 29% (40/140) of the participants randomized. Twenty-two (31%) participants in the primaquine arm reported 45 AEs while 18 (26%) individuals in the no primaquine arm reported 29 AEs (Table 3) Differences in the number of AEs in each MedDRA system order class by treatment arm was not statistically significant (p = 0.34). The majority of AEs were mild, with only one serious AE (SAE) occurring during the trial. The SAE (in the no primaquine arm) was a case of severe renal impairment (estimated glomerular filtration rate, eGFR, of 6 mL/min/1.73 m^2^) detected on day 14 in an adult male with a poorly defined, initially undisclosed medical history of renal impairment. The patient’s eGFR improved to 24 mL/min/1.73 m^2^ by day 42, and 27 mL/min/1.73 m^2^ 3 months later. Eleven moderately-severe AEs (5 in the primaquine arm and 6 in the no primaquine arm) were recorded. Of the five moderately severe AEs in the primaquine arm (1 each of post-dose vomiting, chest pain, headache, urinary tract infection and sexually transmitted infection), the post-dose vomiting was classified as probably associated with primaquine, the headache as possibly associated with primaquine, and the others as unlikely/not related to primaquine. Among those randomized to primaquine, there was no difference between extensive and intermediate CYP2D6 metabolizers in terms of the occurrence of any adverse events (22/61 *vs* 2/6; p = 0.89). No poor metabolizers were enrolled (Table 2).

**Table 3:** Adverse events reported, by treatment arm

	Primaquine	No primaquine
Nervous system disorders: headache 13	6	7
Gastrointestinal: abdominal pain 7, vomiting 3, diarrhoea 1	5	6
Infections: urinary tract infections 5, flu 4, sexually transmitted diseases 3; 1 each eye abscess, tonsillitis, helminths, herpes zoster, malaria recurrence	13	4
Other: general body pains 17, chest pain 3; 1 each cough, dyspnoea, dysmenorrhea, vaginal discharge, dysuria, haematuria, ketonuria, raised JVP, feeling hot, peripheral swelling, eye allergy, ear pain, HIV test positive	21	12

*JVP* jugular venous pressure, *HIV* human immunodeficiency virus

### Therapeutic efficacy of artemether–lumefantrine

Of the 151 participants seen on day 3, none were found to be carrying asexual parasites by microscopy and none met the WHO criteria for early treatment failure. However, by PCR, seven participants (five in the primaquine arm and two in the no primaquine arm) were identified with sub-microscopic asexual parasite carriage on day 3 with only one (in the primaquine arm) having sub-microscopic asexual parasites detectable on day 7. Among the 128 who completed 42-day follow-up, there was one late treatment failure in the primaquine arm (a late parasitological failure with an asexual parasite density of 390/μL but no fever on day 42). Genotype analysis by PCR showed this single treatment failure to be a new infection, giving a PCR-corrected adequate clinical and parasitological response rate of 100%.

### Molecular markers associated with artemether and lumefantrine resistance

All parasite isolates analysed were wild-type (164/164) at the 25 Kelch13 propeller domain alleles assayed, i.e. none of these parasites had mutations associated with artemisinin resistance. However, all 162 isolates had molecular markers associated with reduced lumefantrine susceptibility (wild-type *crt*76LYS and *mdr1*86ASN); one of these isolates (1/165) carried both the wild and mutant *crt*76 alleles. Fortunately, none of the 162 isolates assessed for *mdr1* copy number had the increased copy number associated with lumefantrine resistance.

### Day 7 lumefantrine concentrations

Among the 140 patients randomized, lumefantrine concentrations could be determined for 114 participants (56 from the primaquine arm and 58 from the no primaquine arm). Three concentrations were below the limit of quantification (1 in primaquine arm and 2 in no primaquine arm); these were assumed to be 19.5 ng/mL, i.e. half the lower limit of quantification [62]. The median (interquartile range) lumefantrine concentrations were found to be similar for both primaquine and no primaquine arms [291 (156–559) vs. 343 (180–502) ng/mL, p = 0.95]. Similar results were obtained if the 7 participants who had their pharmacokinetic sample collected outside of the protocol window of day 7 ± 2 days were excluded [329 (179–604) vs. 343 (181–492) ng/mL, p = 0.69].

Day 7 concentrations of below 200 ng/mL previously reported to be sub-optimal and associated with an increased risk of treatment failure [63]. Similar proportions of participants between arms were found to have suboptimal day 7 concentrations 18/56 (32%) in the primaquine arm and 18/58 (31%) in the no primaquine arm [p = 0.90]. Again, similar results were obtained if the 7 participants who had their pharmacokinetic sample collected outside of the protocol window of day 7 ± 2 days were excluded [15/51 (28%) vs. 17/56 (28%); p = 0.92].

## Discussion

This is the first comprehensive controlled study on the risk benefit profile of SLD primaquine as a gametocytocide conducted in a pre-elimination setting in sub-Saharan Africa. Although this study was unable to confirm the efficacy previously demonstrated in larger studies, close monitoring of study participants confirmed the safety and tolerability of SLD primaquine in the local study population, including in 17 patients who carried the *G6PD* A− variant associated with an increased risk of haemolytic anaemia when exposed to 8-aminoquinolines. Unlike previous studies [38, 63–66] that found the percentage decreases in Hb and haemoglobinuria/dark urine to be more likely in individuals receiving low-dose primaquine compared to no primaquine, data from this relatively small but detailed study appears to indicate that marked decreases in Hb were linked to malaria infection itself (with a significant drop in Hb before primaquine randomization and administration on day 3) and *G6PD* variant rather than primaquine treatment. Only two of the previous studies used the 0.25 mg/kg primaquine dose [64, 66] while the others treated patients at a higher dose. Distinguishing malaria-related and primaquine effects is more challenging in studies that administer primaquine on day 0 rather than day 3. All Hb decreases in *G6PD* A− individuals were transient, supporting the Cochrane review conclusion that low-dose primaquine probably has little or no effect on severe haemolysis [63].

The low prevalence of individuals carrying the *G6PD* A− variant, associated with moderate *G6PD* deficiency [18], detected in this study concurs with data from a recent study conducted in neighbouring Limpopo Province [67]. Unlike the current study’s majority Mozambican (Shangaan) population, most individuals in the Limpopo study self-identified as South African Venda; this suggests the A− variant is relatively rare across a range of different ethnic groups in southern Africa. The absence of any variant associated with reduced G6PD activity in Eswatini [68], supports this interpretation.

In line with previous studies [38, 64–66, 69–71], no difference in adverse events between the study arms was observed, with most AEs classified as mild. No difference was observed in the occurrence of adverse events between CYP2D6 extensive and intermediate metabolizers on primaquine; however, no poor metabolizers and few intermediate metabolizers were enrolled so it is not possible to draw any conclusion on the impact of the CYP2D6 phenotype on safety from these data. The single serious adverse event, renal impairment, occurred in a patient who did not receive SLD primaquine and who had a poorly defined history of ‘kidney problems’. This was unfortunately not detected during screening as the patient did not divulge a complete medical history initially and the medical records were not available (as is frequently the case with mobile and migrant populations). The previously-reported increased frequency of anorexia among participants treated with SLD primaquine [63] was not observed in this study. This could be the result of having a study population skewed towards adequately-nourished adults who sought malaria treatment promptly, and the administration of primaquine on day 3 by when malaria symptoms would usually have abated.

Unfortunately, this relatively small study was unable to demonstrate the efficacy of SLD primaquine in reducing gametocyte carriage in an area of extremely low residual transmission. While more than half of the participants carried (mostly sub-microscopic) gametocytes at baseline, very few were still gametocytaemic on day 3 when randomization to the primaquine/no primaquine arms took place. This negligible gametocyte carriage post-ACT may reflect the success of behaviour-change campaigns run by the Mpumalanga Malaria Elimination Programme to encourage early treatment seeking, within 24–48 h of the onset of malaria symptoms, when any gametocytes present are likely to be in the early stages of development and susceptible to artemisinin derivatives. The absence of *CYP2D6* variants with null activity and the low prevalence of variants with slow and intermediate metabolism of inactive primaquine to its active metabolite would infer good primaquine efficacy in the local population. However, the patients enrolled in this study, who were symptomatic and willing to remain in the study area throughout the 6-week follow-up period, are not fully representative of the large mobile and migrant populations that are considered key drivers of ongoing malaria transmission in these areas. These mobile and migrant populations are often asymptomatic and are less likely to be willing to remain in the area for 6-weeks of follow-up [72]. Although dosing on day 3 is preferred for distinguishing primaquine-related adverse effects from malaria related events, it may not be the optimal time of dosing for malaria transmission blocking. Thus, the failure to demonstrate efficacy in this relatively small study should not detract from the potential for SLD primaquine to greatly reduce secondary transmission in the study area and similar areas working towards malaria elimination. Efficacy of SLD primaquine against mature gametocytes and reduction in infectiousness has previously been well established, including in a recent systematic review of larger studies [63]. This showed that the effect on infectiousness precedes the effect of SLD primaquine on gametocyte prevalence, but that there is no evidence yet on whether SLD primaquine could reduce malaria transmission at community level.

In light of the growing concerns over the sustained efficacy of artemether–lumefantrine in the southern African region [73, 74], it was reassuring that this study reported a 100% PCR-corrected adequate clinical and parasitological response. However, almost 90% of the study participants were adults who declared themselves as Mozambican nationals and over two-thirds were found to have two or more *P. falciparum* clones present at baseline, suggesting that acquired premunition may have contributed substantially to this high cure rate. Genotypic analyses in this and other southern Africa studies [73, 75] have revealed strong selection for molecular markers linked with increased tolerance to lumefantrine [76]. Fortunately, an increase in *Pfmdr1* gene copy number (associated with lumefantrine resistance) was not observed in this study and is rare in the region [73, 75, 77]. Although artemisinin-resistant parasites have rapidly spread across the greater Mekong region [78, 79] and are most recently reported in India [80], to date there have been no confirmed reports of artemisinin-resistant parasites becoming established in Africa. Reduced lumefantrine susceptibility would increase pressure on the artemisinin component of ACT, particularly in non-immune individuals. It is, therefore, imperative to enhance anti-malarial resistance surveillance across the southern Africa region to ensure effective treatment policies.

As a limited number of young children were recruited into the trial, the effects of SLD primaquine were not rigorously assessed in this population locally—although there is no reason to believe that these children would respond differently to those studied elsewhere. As primaquine is not yet licensed for use in South Africa, the study’s inclusion and exclusion criteria were stricter than considered necessary by the WHO or that would be used when this intervention is rolled out to advance malaria elimination. However, some evidence of a reassuring safety profile in the broader target population was generated by defining the prevalence of anaemia and *G6PD*/*CYP2D6* genotypes among all those screened. Budgetary constraints restricted drug resistance assessments to a finite number of molecular markers primarily associated with resistance to artemether–lumefantrine. This prevented the generation of detailed drug sensitivity profiles and the possible detection of novel mutations which may affect drug efficacy.

## Conclusion

Safety, tolerability, *CYP2D6* and *G6PD* variant data from this study support the deployment of the WHO-recommended SLD primaquine without *G6PD* testing in South African districts with low-intensity residual transmission aiming to eliminate malaria. Prior to its roll-out, all health care workers should receive comprehensive training on SLD primaquine use with robust pharmacovigilance to strengthen data on primaquine safety in vulnerable populations. The risk of artemisinin resistance spreading from South East Asia to Africa and the strong selection for lumefantrine-tolerant parasites locally and regionally, emphasizes the need for regular and rigorous drug efficacy monitoring.

## Data Availability

All anonymized individual participant data from this study have been shared with the WorldWide Antimalarial Resistance Network (http://www.wwarn.org/working-together/sharing-data).

## Abbreviations

ACT: artemisinin-based combination therapy
AE: adverse event
*CYP2D6*: cytochrome P450 2D6
*crt*76: codon 76 of the *P. falciparum* chloroquine resistance transporter gene
DBS: dried blood spot
eGFR: estimated glomerular filtration rate
*G6PD*: glucose-6-phosphate dehydrogenase
Hb: haemoglobin
*mdr*86: codon 86 of the *P. falciparum* multi-drug resistance gene
PCR: polymerase chain reaction
RDT: rapid diagnostic test
RT-PCR: reverse transcriptase-polymerase chain reaction
SAE: severe adverse event
SLD: single low-dose
qPCR: quantitative polymerase chain reaction
